# Application of Chromatographic and Spectroscopic-Based Methods for Analysis of Omega-3 (ω-3 FAs) and Omega-6 (ω-6 FAs) Fatty Acids in Marine Natural Products

**DOI:** 10.3390/molecules28145524

**Published:** 2023-07-19

**Authors:** Abdul Rohman, Irnawati, Anjar Windarsih, Florentinus Dika Octa Riswanto, Gunawan Indrayanto, Nurrulhidayah A. Fadzillah, Sugeng Riyanto, Nor Kartini Abu Bakar

**Affiliations:** 1 Halal Center, Universitas Gadjah Mada, Yogyakarta 55281, Indonesia; 2 Department of Pharmaceutical Chemistry, Faculty of Pharmacy, Universitas Gadjah Mada, Yogyakarta 55281, Indonesia; 3 Faculty of Pharmacy, Universitas Gadjah Mada, Yogyakarta 55281, Indonesia; irnawati.vhina@gmail.com; 4 Study Program of Pharmacy, Faculty of Pharmacy, Halu Oleo University, Kendari 93232, Indonesia; 5 Research Center for Food Technology and Processing (PRTPP), National Research and Innovation Agency (BRIN), Yogyakarta 55861, Indonesia; anjarwindarsih2@gmail.com; 6 Department of Chemistry, Faculty of Science, Universiti Malaya, Kuala Lumpur 50603, Malaysia; kartini@um.edu.my; 7 Department of Pharmacy, Faculty of Pharmacy, Campus III Paingan, Sanata Dharma University, Yogyakarta 55282, Indonesia; dikaocta@usd.ac.id; 8 Faculty of Pharmacy, Universitas Surabaya, Surabaya 60286, Indonesia; gunawanindrayanto@yahoo.com; 9 International Institute for Halal Research and Training, International Islamic University Malaysia, Kuala Lumpur 53100, Malaysia; nurrulhidayah@iium.edu.my; 10 Study Program of Pharmacy, Faculty of Health Sciences and Pharmacy, Universitas Gunadarma, Jakarta 16451, Indonesia; sugeng_riyanto@yahoo.com

**Keywords:** spectroscopic techniques, omega-3 fatty acids, ω-6 FAs, chemometrics, quality control, chromatographic methods

## Abstract

Omega-3 fatty acids v(ω-3 FAs) such as EPA (eicosapentaenoic acid) and DHA (docosahexaenoic acid) and omega-6 fatty acids (ω-6 FAs) such as linoleic acid and arachidonic acid are important fatty acids responsible for positive effects on human health. The main sources of ω-3 FAs and ω-6 FAs are marine-based products, especially fish oils. Some food, supplements, and pharmaceutical products would include fish oils as a source of ω-3 FAs and ω-6 FAs; therefore, the quality assurance of these products is highly required. Some analytical methods mainly based on spectroscopic and chromatographic techniques have been reported. Molecular spectroscopy such as Infrared and Raman parallel to chemometrics has been successfully applied for quantitative analysis of individual and total ω-3 FAs and ω-6 FAs. This spectroscopic technique is typically applied as the alternative method to official methods applying chromatographic methods. Due to the capability to provide the separation of ω-3 FAs and ω-6 FAs from other components in the products, gas and liquid chromatography along with sophisticated detectors such as mass spectrometers are ideal analytical methods offering sensitive and specific results that are suitable for routine quality control.

## 1. Introduction

**Figure 1:**
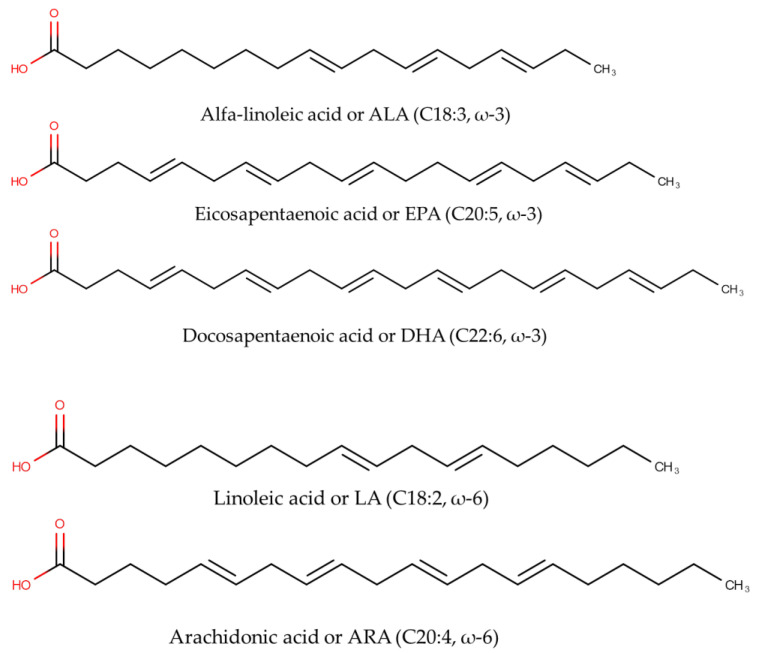
The chemical structures of omega-3 fatty acids including alpha-Linolenic acid (C18:3, ω-3), eicosapentaenoic acid or EPA (C20:5, ω-3), and docosahexaenoic acid or DHA (C22:6, ω-3), as well as omega-6 fatty acids including linoleic acid or LA (C18:2, ω-6) and arachidonic acid or ARA (C20:4, ω-6).

Fish oils are good sources of polyunsaturated fatty acids (PUFA) including omega-3 fatty acids (ω-3 FAs) and omega-6 fatty acids (ω-6 FAs). In terms of chemical structures, ω-3 FAs and ω-6 FAs can be differentiated from the first double bond locations, counting from the terminal of methyl groups of the corresponding fatty acid. In ω-6 FAs, the first double bond is located between the sixth and seventh carbon atoms, while for ω-3, the first double bond is located between the third and fourth carbon atoms [1]. Alpha-Linolenic acid or ALA (C18:3, ω-3), eicosapentaenoic acid or EPA (C20:5, ω-3), docosapentaenoic acid or DPA (20:5 n-3), docosahexaenoic acid or DHA (C22:6, ω-3), tetracosapentaenoic acid or TPA (C24:5 n-3), and tetracosahexaenoic acid or THA (C24:6n-3) are among ω-3 FAs, while linoleic acid or LA (C18:2, ω-6) and arachidonic acid or ARA (C20:4, ω-6) are example of ω-6 FAs. The chemical structures of selected ω-3 FAs and ω-6 FAs are depicted in Figure 1.

In marine oils, the most dominant ω-FAs are DHA, ARA, and EPA. Linolenic and linoleic acids are essential FAs needed by the human body and contain double bonds that are not synthesized in the human body [2]. ω-3 FAs are believed to contribute positive potentials to human health. The beneficial health effects of ω-3 FAs, especially EPA and DHA, were firstly described in the Greenland Eskimos, who consumed a high seafood diet and exhibited low rates of coronary heart disease, type I diabetes mellitus, asthma, and multiple sclerosis. Since this observation, the advantageous effects of ω-3 FAs have been widely explored. The consumption of foods containing EPA and DHA is associated with low risk of cardiovascular attack [3]. EPA and DHA are essential for proper fetal development and brain development and for lowering depression risk [4]. It is believed that a diet rich in ω-6 FAs could promote the pathogenesis of some chronic inflammatory diseases. These beneficial effects have attracted scientists to explore the natural sources of ω-3 and ω-6 FAs. A well-balanced ω-3/ω-6 FAs ratio is reported to be proven for promoting health and preventing disease formation, mostly in the Mediterranean diet. In addition, the balance of ω-3 and ω-6 FAs is an important determinant in decreasing the risks of coronary heart disease (CHD), cancer, diabetes, arthritis, hypertension, and other neurodegenerative diseases. As a consequence, some research has reported this ratio value. The ratio of ω-6/ω-3 from 3:1 to 4:1 could prevent pathogenesis of various diseases [5].

**Figure 2:**
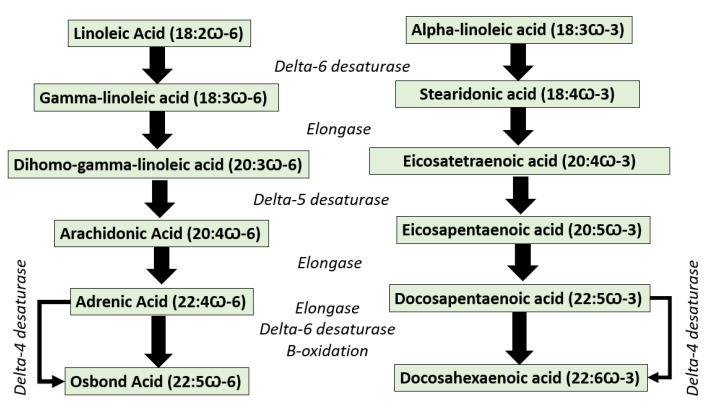
The conversion pathway of omega-3 fatty acids represented by osbond acid and omega-6 fatty acids represented by docosahexaenoic acid [11].

EPA and DHA cannot be synthesized in the human body de novo; therefore, human and mammals should obtain them from the diet. However, both EPA/DHA and other n-3 long-chain poly-unsaturated fatty acids can be synthesized from their precursor of alpha-linolenic acid (ALA, 18:3 n-3) through the desaturation and elongation of EPA. Unfortunately, the conversion rate of ALA into EPA/DHA is very low because of the limited activity of the ∆6-desaturase enzyme [6] (Figure 2). Some fish species have elongase and desaturase enzymes, which enable them to convert linoleic acid (LA, 18:2 n-6) into ARA and ALA into EPA and DHA [7]. Thus, EPA/DHA must be provided favorably from the diet, especially from the main source of marine-based foods [8]. According to the Food and Agriculture Organization (FAO), the recommended daily intake of EPA + DHA is set between 100 and 250 mg for children up to 10 years, 250 mg for healthy adults, and 300 mg for pregnant women (of which at least 200 mg must be DHA). On the other hand, arachidonic acid, an n-6 polyunsaturated 20-carbon fatty acid formed by the biosynthesis of linoleic acid, was reported to play important roles in infant development [9]. For this reason, the evaluation of ω-FAs is very important to confirm their contents in food and supplement products [10].

## 2. Chemometrics

**Figure 3:**
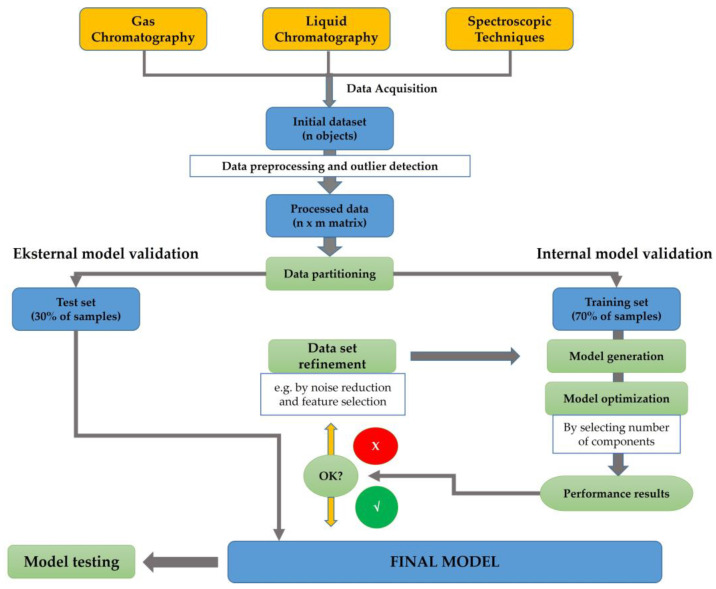
The detailed workflow of analytical methods and chemometrics techniques used for analysis of omega-3 (ω-3) or omega-6 (ω-6) fatty acids using spectroscopic and chromatographic-based methods [20].

Chemometrics can be described as the science implementing statistical and mathematical techniques to obtain relevant information related to process, design, and optimal selection by analyzing chemical data [12,13]. In the last two decades, chemometrics has been widely applied in the field of chemistry to enhance analytical methods such as spectroscopy and chromatographic-based methods [14,15]. Chemometrics of exploratory data analysis of Principal Component Analysis (PCA) was applied for authenticating several oils using an open source chemometrics software [16]. Another study has successfully performed the classification of marine fish surimi according to the species, namely white croaker surimi (*Argyrosomus argentatus*), hairtail surimi (*Trichiurus haumela*), and red coat surimi (*Nemipterus virgatus*) [17]. In order to enhance the quality of the authentication purposes, chemometrics of pattern recognition were widely applied. Unsupervised pattern recognition of PCA and supervised pattern recognition of linear discriminant analysis (LDA) and Soft Independent Modeling of Class Analogies (SIMCA) have been generated to investigate the potential substitution of valuable fish species with cheaper materials [18]. A multivariate calibration technique of partial least squares regression (PLSR) has been successfully developed to build prediction models for determining fatty acids content (%) from eight fish oil samples [19]. Figure 3 presents the scheme of chemometrics application for analyzing omega-3 or omega-6 fatty acids using spectroscopic and chromatographic-based methods.

A set of initial data, which are collected from analytical instruments such as spectrophotometer and/or chromatography, could be used for chemometrics analysis. At first, several data pre-processing and outlier detections can be applied to support the subsequent analytical modeling [21]. In the case of supervised pattern recognition modeling, a matrix of data achieved from the pre-processing stage has been divided into two groups consisting of the training set (70% of the data) and test set (30% of the data). This partition will enable the implementation of internal model validation, cross validation, and external validation of the model [22]. The generation of the model followed by the model optimization by components selection are then executed. Performance quality for model accuracy can be evaluated by assessing the determination coefficient of the calibration model (R_cal_^2^) and the determination coefficient of cross-validation (R_CV_^2^), whereas the difference or error between the actual and predicted values of the model can be evaluated by root mean square error of calibration (RMSEC) and root mean square error of cross-validation (RMSECV) [23]. It can be noted that the data refinement can be applied to improve the model quality. The final chemometrics model is generated by considering the external validation using test set data. The quality of the prediction should be evaluated using the determination coefficient of validation (R_val_^2^) and root mean square error of calibration (RMSEP). Further, the final chemometrics techniques with appropriate features can be applied for sample evaluation.

## 3. Official Methods of Analysis of ω-3 FAs and Related Compounds

In the current USP-NF online, three ω-3 FA compounds have been described, i.e., ω-3 free fatty acids [24], ω-3 acid triglycerides [25], and ω-3-acid ethyl esters [26]. Ω-3 free fatty acids are a mixture of ω-3 acids in their free form. They consist mainly of alpha-linolenic acid (C18:3 n-3), moroctic acid (C18:4 n-3), eicosatetraenoic acid (C20:4 n-3), eicosapentaenoic acid (EPA) (C20:5 n-3), heneicosapentaenoic acid (C21:5 n-3), docosapentaenoic acid (C22:5 n-3), and docosahexaenoic acid (DHA) (C22:6 n-3) [24]. Ω-3 acid triglycerides are a mixture of mono, di, and tri-esters of ω-3 acids with glycerol containing mainly tri-esters and obtained either by esterification of concentrated and purified ω-3 acids with glycerol or by transesterification of the ω-3 acid ethyl esters with glycerol [25].

Omega-3-acid ethyl esters are a mixture of ethyl esters, principally the ethyl esters of eicosapentaenoic acid (EPAee) (C20:5 n-3, EE) and docosahexaenoic acid (DHAee) (C22:6 n-3, EE). They may also include ethyl esters of alpha-linolenic acid (C18:3 n-3, EE), moroctic acid (C18:4 n-3, EE), eicosatetraenoic acid (C20:4 n-3, EE), heneicosapentaenoic acid (C21:5 n-3, EE), and docosapentaenoic acid (C22:5 n-3, EE). Tocopherol may be added as an antioxidant [26].

The content of EPA, DHA, and total ω-3 free fatty acids are determined using GC equipped with FID (270°), column: 0.25-mm × 25-m; coated with a 0.20-µm film of phase G16 (Carbowax 20M), injection port: 250°, split ratio: 200/1 temperature programmed: initial: 170° (2 min) then raise to 240° (3°/min, 2.5 min). For verifying the methods, two SSTs (standard solution tests) are used; SST 1 consists of USP ω-3 free fatty acid RS, while SST 2 consists of DHA methyl ester and tetracos-15-enoic acid methyl ester. USP EPA RS and USP DHA RS are used as the standard, and USP methyl tricosanoate RS is used as IS. Detailed preparations of SST solutions and samples can be referred to in this official method [24].

Acceptance criteria: RRt (relative retention time) of alpha-linolenic acid (C18:3 n-3;0.564), moroctic acid (C18:4 n-3; 0.592), eicosatetraenoic acid (C20:4 n-3; 0.776), EPA (C20:5 n-3; 0.793), heneicosapentaenoic acid (C21:5 n-3; 0.887), and docosapentaenoic acid (C22:5 n-3; 0.973) are calculated against Rt (retention time) of DHA (C22:6 n-3; 1.00). Chromatogram of sample should be similar to SST 1. EPA should be NLT 45% and NMT 65%, DHA NLT 10% NMT 30%, and total ω-3 free fatty acids NLT 80%. [24]

Unfortunately, this current USP-NF online (https://online.uspnf.com/uspnf, accessed on 28 May 2023) does not describe the method for determination of similarity of the chromatograms. Alaerts et al. described the application of the correlation coefficient **r** and the congruence coefficient **c** for the determination of the similarity [27].

The following official procedure of the general chapter <401> of USP 46-NF 41 may be used for the determination of eicosapentaenoic acid (EPA) (C20:5 n-3), docosahexaenoic acid (DHA) (C22:6 n-3), and total ω-3 FAs obtained from fish, plant, or microbial sources in bulk oils and encapsulated oil, either as triglycerides or as ethyl esters. The term “triglyceride” is applicable to algal oils, fish oils, fish liver oils, and products containing ω-3 FAs in triglyceride form [28]. If using split injection, the GC-FID method is identical to [16], if using splitless injection, the conditions have modified as follows: injection port: 90–250°, column temperature programmed: 90° (2 min) raise to 170° (30/min, 3.0 min), then raise to 240° (3°/min, 2 min). Concentrations of EPA, DHA, and total ω-3 FAs (for triglycerides or ethyl ester) are estimated. Detailed methods including sample preparations, SSTs, and acceptance criteria have been described in this general chapter [28].

The official GC method of the general chapter <401> of USP 46-NF 41 [28] has been applied for the determination of EPA, DHA, and total ω-3 FAs in the monograph of ω-3 acid triglycerides [25], ω-3 acid ethyl esters [23,26], fish oil containing ω-3 FAs [29], and other related monographs including materials from plants. In the current USP-NF online https://online.uspnf.com/uspnf (accessed on 29 May 2023), 15 monographs describe the methods of analysis of ω-3 FAs and related compounds. SSTs and acceptance criteria can be referred to in the respective monographs. Verification or validation methods should be performed prior to routine application of the official methods in the QC laboratory of the pharmaceutical industry. If the results of the SSTs can fulfil the criteria that are described in the respected monographs, there is no need to perform full validation methods [30,31].

## 4. Chromatographic-Based Methods for Analysis of ω-3 FAs and ω-6 FAs

Due to their capacity as separation techniques, chromatographic-based methods offered excellent analytical methods for analysis of ω-3 FAs and ω-6 FAs in marine products. Currently, the types of chromatograph instruments along with their components, mainly columns and detectors, are available in the laboratories, therefore, the routine analysis of fatty acids can be carried out efficiently and effectively [32]. Chromatographic methods can be used for separation, identification, and quantification of these fatty acids with reliable results. Gas chromatography using detectors such as a flame ionization detector (GC-FID) and mass detector (GC-MS) are the most common chromatographic techniques used for determination of ω-3 FAs and ω-6 FAs. Liquid chromatography tandem with mass spectrometry (LC-MS or LC-MS/MS) is also an effective analytical technique for the analysis of omega fatty acids without the derivatization step [33].

### 4.1. Gas Chromatography for Analysis of ω-3 FAs and ω-6 FAs

Among analytical methods used for fatty acids analysis, chromatographic-based techniques, mainly gas chromatography, have been extensively reported for analysis of fatty acids including ω-3 FAs and ω-6 FAs. In addition, GC also provides a sensitive, accurate, precise, rapid, and reproducible analysis with efficient separation due to the low mass transfer compared to liquid chromatography.

Gas chromatography (GC) is a chromatographic separation technique based on the difference in the distribution of organic compounds between two non-miscible phases in which the mobile phase is a carrier gas moving through or passing the stationary phase contained in a column. GC is appropriate for analyzing any organic compounds or their derivatives, which are volatilized under the temperatures employed. Mainly, GC is based on the mechanisms of adsorption or mass distribution [34]. The common carrier gas is helium, nitrogen, or hydrogen, and it depends on the column and detector in use. The flow of carrier gas through the column and the detector is performed at a controlled rate or pressure [34,35]. A brief discussion regarding general GC system instrumentation has been previously published [36], while discussion on gas chromatography–mass spectrometry (GC-MS) systems can be referred to in previous published papers [37,38].

Injection may be performed either into a vaporization chamber, which may be equipped with a stream splitter, or directly at the head of the column using a syringe or an injection valve [34]. Today, the most used injectors for capillary GC fall into one of four techniques, i.e., split, splitless, on-column injection, and programmed-temperature vaporizers (PTV) [39]. The liquid stationary phases used in GC are contained in a column that comprises either a capillary column (at least 5 m, 0.1–0.53 mm i.d.) whose stationary phase may be a solid coating on the inner surface (0.1–5.0 µm film) of the column (e.g., macrogol 20,000) or a liquid deposited on the inner surface (e.g., dimethylpolysiloxane); in the latter case, it may be chemically bonded to the inner surface and a column packed (1–3 m, 2–4 mm i.d.) with the stationary phase that may be a solid phase (e.g., alumina, silica) or an inert solid support (usually a porous polymer impregnated or coated with a liquid) [34,35].

Two distinct types of detectors are used in GC, namely mass-dependent or concentration-dependent. A mass-dependent detector responds to mass per unit time entering the detector, not mass per unit volume. Mass-dependent detectors are destructive, and the compounds eluting from the column are chemically modified or destroyed in the detector (e.g., FID, flame photometric detector (FPD), nitrogen chemiluminescence detector (NCD), mass spectrometer (MS)). Concentration-dependent detectors are non-destructive and can therefore be used in series with other detectors (e.g., thermal conductivity detector (TCD), electron capture detector (ECD), Fourier Transform Infrared (FT-IR)) [40]. Detectors for GC can be also classified based on their selectivity. TCD is a universal detector that can respond to all compounds except the carrier gas. Most common GC detectors fall into the selective designation, e.g., FID for all organic compounds that contain C–C and C–H bonds, ECD for organic compounds that contain halogen and phosphorous groups, FPD for S and P compounds, NCD for N compounds, and NPD for pesticides. In addition, MS and FT-IR detectors can be used for identifying organic compounds in samples by comparing their spectra to authentic standards and/or libraries [41].

GC can be applied for qualitative and quantitative analysis. Quantitative analysis can be performed using direct calibration (using external and internal standard methods) and standard addition methods [34,41]. Fingerprint analysis can be performed for the identification and characterization of plant/herbal materials and related products [42]. Identification of organic compounds in herbal or plant materials can be performed using combination GC-MS or GC-HR-MS-MS and many free online MS databases. To have reliable results of analysis, all methods (qualitative and quantitative) should be properly validated prior to routine application. Detailed validation methods for chromatography analysis have been reviewed and discussed by previous publications [43,44].

The advancements in GC instrumentation coupled with data processing have led to the evolution of this method as a reference method for analysis of fatty acids in marine organisms. However, in some cases, the extensive sample preparation including extraction and derivatization steps must be done before being subjected to GC analysis [45]. Fatty acids are non-volatile enough; therefore, the derivatization of fatty acids is required. In GC, the derivatization step is required when the analytes are non-volatile, thermally labile, and low sensitivity. Methylation of FAs is the most common method for derivatization of FAs to provide volatile fatty acid methyl esters (FAMEs). Many methylation techniques are reported in the scientific literature, and among them, acid-catalyzed methylation, alkaline-catalyzed methylation, methylation using boron trifluoride (BF3), methylation with diazomethane, and silylation are the most used [46]. Extraction of fatty acids and other lipid components was typically carried using different methods (direct extraction, maceration, Soxhlet extraction, Folch, Bligh–Dyer) employing organic solvents with different polarities [47].

GC-FID has been developed and validated for quantitative analysis of ω-3 FAs of DHA and EPA in raw and cooked fish [48]. Fatty acids were derivatized as fatty acid methyl esters (FAMEs), and the derivatized fatty acids were subjected to GC-FID analysis. The column used is a capillary column of a GC HP-88 column (60 m length, 0.25 mm ID, 0.2 µm DF) with a polar stationary phase. The validation of GC-FID followed the guidance from the International Conference on Harmonization (ICH) by determining and evaluating some characteristics of performance including linearity with r-value > 0.9995, precision with RSD ≤ 2%, accuracy with recovery percentage of >95%, specificity with good peaks separation, and sensitivity with low detection limits. All validation parameters meet acceptance criteria set by ICH requirements. The developed analysis was successfully employed for determination of EPA and DHA in fish samples. GC-FID is also successful for determination of omega-3 and omega-6 fatty acids including EPA and DHA by applying two different cyanopropyl silicone capillary columns (DB-225ms and DB-23). The method is valid based on ICH requirements [46].

GC-MS, the combination of two powerful instruments, namely GC for separation and MS for detection, has become a popular method for the confirmation and quantitative analysis of ω-3 FAs (EPA/DHA) in supplement capsules containing fish oils. Quantification of EPA/DHA was carried out using selective ion mode (SIM) using *m*/*z* 79 as the ion fragment EPA/DHA. Fatty acids were released from triglycerides using KOH (potassium hydroxide)-methanolic solution (followed by esterification with BF3-methanol solution to provide FAMEs). The method was validated according to the ICH (International Council for Harmonization), and all validation parameters fit with acceptance criteria set by ICH requirements. Limit of detection (LoD) values obtained were 0.08 ng and 0.21 ng for EPA and DHA, respectively, with RSD values of intra-day and inter-day variations of <0.59% and 1.00% for EPA and <1.08% and 1.05% for DHA. The percentages of average recoveries for EPA and DHA were 100.50% and 103.83%, respectively. The validated method was successfully used for analysis of EPA and DHA contents in commercial capsules containing fish oils. There is no correlation between the contents of EPA/DHA and the price of supplement capsules [49]. GC-MS was also applied for analysis of ω-3 (ALA, EPA, DHA, DPA) and ω-6 FAs (dihomo-linoleic acid or DGLA and ARA) with valid results [50]. Table 1 lists the application of GC-FID and GC-MS for analysis of ω-3 and ω-6 FAs in fish oils contained in different marine products.

### 4.2. Liquid Chromatography for Analysis of ω-3 and ω-6 FAs

Traditionally, ω-3 FAs and ω-6 FAs were determined using GC-FID and GC-MS, in which both analytes of ω-3 FAs and ω-6 FAs must be extracted from the marine samples to release them from their triglycerides, and FAs were derivatized to their volatile methyl esters (FAMEs) prior to analysis by GC. Even though GC-FID or GC-MS are the standard methods for analysis of ω-3 FAs and ω-3 FAs, they are very tedious and time-consuming since these methods comprise several steps including extraction, hydrolysis, and derivatization before being subjected to GC-FID/GC-MS. In addition, the use of high temperature during GC measurement can affect the stability of FAs. For this reason, a greener method of analysis based on HPLC, which does not involve the complex step, should be used. Direct HPLC-UV detection is limited for determination of ω-3 FAs and ω-6 FAs since FAs lack chromophore groups requiring low wavelengths, which limits the solvent selection and increases the likelihood of matrix interference. The combination of the dual-gradient HPLC method and charged aerosol detection has enabled the determination of ω-3 FAs and ω-3 FAs in a single analysis without derivatization of analytes as in GC-FID/GC-MS.

- Liquid chromatography (LC) is a chromatographic separation technique based on the difference in the distribution of species between two non-miscible phases in which the mobile phase is a liquid that eluted through a stationary phase contained in a column. The term LC is synonymous with high-pressure liquid chromatography (HPLC) [35,59]. Particle diameter of the stationary phase for classical HPLC is about 2–10 µm; if the particle diameter is less than 2 µm, the term becomes ultra-high-performance liquid chromatography (UHPLC) [60]. A liquid chromatograph comprises (1) a pumping system, (2) an injector, (3) a chromatographic column (a column temperature controller may be used), and (4) one or more detectors and data acquisition systems [59].

Depending on the type of interaction of the sample molecules with both mobile and stationary phases, different types of interactions can be possible in LC: adsorption chromatography, partition chromatography, ion exchange chromatography, affinity chromatography, and size exclusion chromatography [60]. Stationary phases used for LC are silica or alumina, a variety of chemically modified supports prepared from polymers, silica or porous graphite, resins or polymers with acidic or basic groups, porous silica or polymers, and specially modified stationary phases for the separation of enantiomers (chiral chromatography) [59].

Mobile phases are a solvent or a mixture of solvents, as defined in the individual monograph of the Pharmacopeias. For normal-phase LC, low-polarity organic solvents are generally employed. In reversed-phase LC, aqueous mobile phases, usually with organic solvents and/or modifiers, are employed. Mobile phases may contain other components, for example, a counter-ion for ion-pair chromatography or a chiral selector for chiral chromatography using an achiral stationary phase. The technique of continuously changing the mobile phase’s composition during the chromatographic run is called gradient elution or solvent programming. Mobile phases should be filtered to remove particles greater than 0.45 µm in size (or greater than 0.2 µm when the stationary phase is made of sub-2.0 µm particles and when special detectors, e.g., light-scattering detectors, are used) [35,59].

Many detectors can be used for LC; these include UV-Vis, DAD, fluorescence spectrophotometers, RI, ED, CAD, MS or MS/MS, and MALS [59]. In chemical or pharmaceutical analysis, LC can be applied for identification and purity tests and quantitative analysis (assay) using absolute curve calibration methods and internal standardization methods [61]. Qualitative identification and purity tests of plant/herbal materials and related products using fingerprints have been previously reviewed and discussed [42]. Detailed theory, method development, optimizations, and troubleshooting using LC methods have been described and discussed [60]. Identification of organic compounds or metabolites in plant/herb materials can be performed using combination LC-HR-MS/MS and many online MS databases. Reliable data can be only obtained if all methods used are validated prior to applications; for detailed discussion regarding this matter, refer to the previous publications by Riswanto et al. and Irnawati et al. [14,16]. If the recovery of the method using LC-MS/MS is not acceptable, the matrix effect should be evaluated [62]. Table 2 compiles the application of HPLC and LC-MS for analysis of ω-3 and ω-6 FAs in marine products.

**Figure 4:**
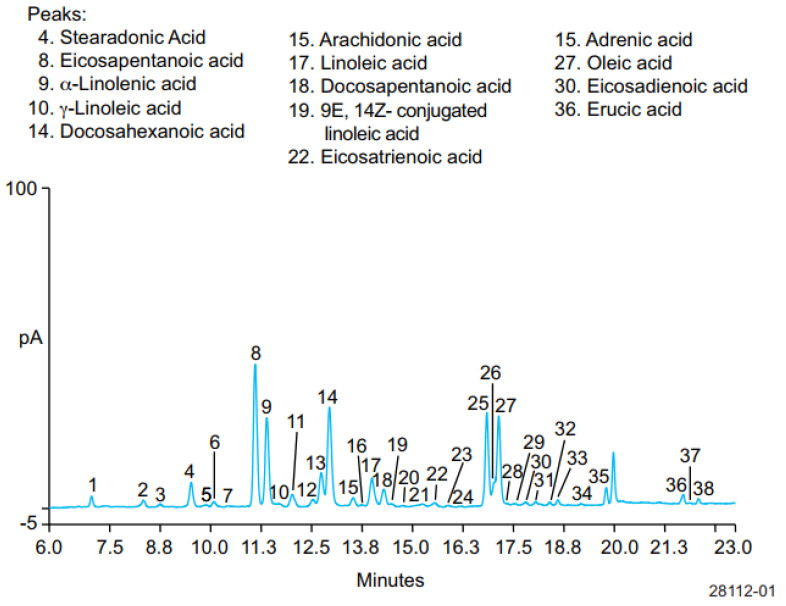
Separation of stearidonic acid (SDA), EPA, ALA, DHA, docosapentaenoic acid or DPA, and Eicosatrienoic acid (ETA); five ω-6 FAs, γ-linolenic acid (GLA), ARA, linoleic acid (LA), adrenic acid, and eicosadienioic acid (EDA); and two omega-9 fatty acids (oleic and erucic acids) [73]. Figure was obtained from Thermo Scientific.

HPLC using a charged aerosol detector (CAD) has been used for separation and quantitative analysis of six ω-3 FAs, SDA, EPA, ALA, DHA, DPA, and ETA, five ω-6 FAs, GLA, ARA, LA, adrenic acid, and eicosadienioic acid (EDA), and two omega-9 fatty acids, oleic and erucic acids, in hydrolyzed fish oil-based commercial supplements. CAD is mass sensitive, which can be added to HPLC and other LC system platforms, providing the most consistent response for semi-volatile and non-volatile analytes. This detector works by charging the analytes, and the charged analytes were separated using the column Acclaim™ C30 (250 × 3 mm, 3 µm) at a temperature of 30 °C. The mobile phase used consisted of mobile phase A of water:formic acid:mobile phase B (900:3.6:100 *v*/*v*/*v*) with mobile phase B of acetone:acetonitrile:tetrahydrofuran:formic acid (675:225:100:4 *v*/*v*/*v*/*v*) delivered in a gradient manner. Figure 4 exhibits the separation profiles of ω-FAs in hydrolyzed fish oil. All ω-3 FAs and ω-6 FAs were well separated with good efficiency and resolution. In addition, the developed method is valid and meets ICH requirements as indicated by acceptable criteria of performance characteristics such as linearity and recovery. LoD values were in the range of 1–10 ng. Using this method, the relative levels of ω-3 FAs, ω-6 FAs, and omega-9 FAs in fish oils were of 92.0%, 3.2%, and 4.5%, with the ratio of ω-3 to ω-6 being 29.0 [73].

HPLC coupled with DAD (diode array detector) has been successfully applied to analyze EPA and DHA in marine diatom biomass. Samples of biomass were extracted using different extraction methods such as Soxhlet and supercritical fluid extraction methods. After extraction, the obtained extracts were subjected to derivatization using 2,4-dibromoacetophenone and triethylamine. Analysis was performed using the HALO C18 column, and the elution was carried out using a mobile phase of acetonitrile (85%) and water (15%) in isocratic mode at flow rate of 0.5 mL/min. The sample was injected at 5 µL. During the analysis, the column temperature was set at 35 °C, and the detection of EPA and DHA was performed using DAD at a wavelength of 254 nm. Results showed that the most EPA and DHA was obtained from reflux extraction (EPA = 63.746 mg/g, DHA = 1.379 mg/g). The second most EPA and DHA was obtained from Soxhlet extraction (EPA = 39.729 mg/g, DHA = 1.059 mg/g), followed by the supercritical fluid extraction method (EPA = 33.701 mg/g, DHA = 0.455 mg/g) [74].

Liquid chromatography tandem with mass spectrometry (LC-MS/MS) using multiple reaction monitoring (MRM) mode has been reported for analysis of ω-3 and ω-6 FAs ALA, DHA, LA, ARA, and EPA. LC-MS/MS offers a direct method for analysis of fatty acids without derivatization steps. The ion transitions used are at *m*/*z* values of 277.25→259.15 (ALA), 327.30→283.35 and 327.30→229.35 (DHA), 279.20→261.25 (LA), 303.30→259.35 and 303.30→205.35 (ARA), as well as 301.25→257.30 and 301.25→203.30 (EPA) [75]. Liquid chromatography tandem mass spectrometry (LC-MS/MS) has also been utilized for analysis of EPA and DHA in biological samples. The reverse-phase chromatography was applied using a C18 column (50 mm, 4.6 mm, 5 µm). During the analysis, the temperature of the column was maintained at 40 °C, whereas the sampler temperature was 10 °C. Elution was performed in isocratic mode using acetonitrile:ammonium formate buffer (10 mM) containing 0.05% formic acid as the mobile phase. The EPA and DHA were analyzed in a positive ionization mode using a triple quadrupole mass spectrometer. The multiple reactions monitoring (MRM) transition used for quantification was 331.30→285.00 for ethyl eicosapentaneoate, 336.30→285.00 for ethyl eicosapentaneoate D5, 357.30→131.00 for ethyl docosahexenoate, and 362.00→131.00 for ethyl docosahexenoate D5. It showed that the LC-MS/MS method could be used to measure EPA and DHA in a simple way. In addition, it can be applied in samples with high interferences [32].

## 5. Molecular Spectroscopic Methods for Analysis of ω-3 FAs and ω-6 FAs

Molecular spectroscopy is the collection of analytical methods based on the interaction between electromagnetic radiations in a certain energy with samples in the molecular levels. UV-Visible, Raman, Infrared (near, mid, and far), and nuclear magnetic resonance are among the molecular spectroscopic techniques widely used for analysis of fatty acid compositions. The different concentrations and functional groups present in ω-3 FAs and ω-6 FAs are main factors contributing to the different spectra profiles [76]. Combined with chemometrics techniques, molecular spectroscopic techniques, mainly Raman and FTIR, have been widely applied for the prediction of ω-3 FA and ω-6 FA levels in marine products [77,78].

FTIR spectroscopy in combination with chemometrics of multivariate calibrations has been developed and validated for analysis of total ω-3 FAs and ω-6 FAs in adipose tissue of rainbow trout (*Onchorhynchus mykiss*) fish. The individual ω-3 FAs (APA, EPA, and DHA) and individual ω-6 FAs (ALA and ARA) were determined using GC-FID. The multivariate calibrations of PLS and ridge regression methods were used for constructing the quantitative model of predicting ω-3 FAs and ω-6 FAs applying the absorbance values at combined wavenumber regions of Raman spectra at 550–1800 and 2610–3100 cm^−1^. Raman spectra were previously subjected to pre-processing using standard normal variate (SNV) transformation to eliminate Raman spectral variations. The processed Raman spectra were used for PLS and ridge regression methods (RRM). PLS and RRM are designed to estimate linear prediction scores when the number of predicting variables (Raman spectra at selected wavenumbers) exceeds the training sample size. RRM introduces a shrinkage parameter intended to find the best compromise between prediction and variance. The cross-validation was employed to compare the prediction models as represented by RMSEP and RMSECV values. In cross-validation models, the actual values of total ω-3 FAs and ω-6 FAs as determined by GC-FID and FTIR predicted values provided an R^2^ of 0.66 and 0.86, respectively. For individual ω-3 FAs, ALA, EPA, and DHA were found to have good R^2^ values (0.82, 0.76, and 0.81, respectively). This indicated that the combination of Raman and PLS-RRM is suitable for analysis of individual ω-3 FAs and total ω-6 FAs but is not accurate for prediction total ω-3 FAs [79].

Previous studies also reported the employment of FTIR spectroscopy combined with PLS for prediction of the levels of EPA, DHA, and total ω-3 FAs in micro-encapsulated fish oil supplements. The supplement powders were directly subjected to the attenuated total reflectance (ATR) accessory, and their FTIR spectra were recorded at 4000–500 cm^−1^ in absorbance mode. The chemometrics of exploratory data analysis of PCA was used to identify the principal components (PC), which are linear combinations of original variables (absorbance values at certain wavenumbers capable of identifying the wavenumbers directly proportional to levels of EPA, DHA, and total ω-3 FAs). The PCA matrix plot of the spectral results reveals an increased intensity of peaks at wavenumbers of 2923, 1744, and 1146 cm^−1^, therefore, the combined wavenumbers region of 3100–2800 and 1800–950 cm^−1^, which represented the best model for every target fatty acid, was optimized and used for quantitative modeling. Using multivariate calibration of PLS, a good correlation existed between actual values of EPA, DHA, and total ω-3 FAs as determined using GC-FID (x-axis, in mg/g) and FTIR predicted values (y-axis, in mg/g) with the equations of:

y = 1.0058x + 0.3070 with R^2^ of 0.9878, RMSE of 1.5931 (EPA),

y = 1.0058x + 0.3070 with R^2^ of 0.9875, RMSE of 1.0926 (DHA), and

y = 1.0058x + 0.3070 with R^2^ of 0.9875, RMSE of 1.0926 (total ω-3 FAs).

The high R^2^ and low values of RMSE indicated that FTIR spectroscopy at selected wavenumbers regions offer accurate and precise analytical methods for the prediction of EPA, DHA, and total ω-3 FAs. This developed method could be an alternative technique to GC-FID [77].

Raman spectroscopy in combination with chemometrics of standard normal variate (SNV) and multivariate calibration of PLS has been successfully used for the prediction of EPA, DHA, and total ω-3 FAs. Some regions of Raman spectra were subjected to optimization to provide the best relationship models between actual values of EPA, DHA, and total ω-3 FAs as determined using GC-FID and FTIR predicted values. The regions capable of providing the highest R^2^ values and the lowest values of RMSEC and RMSEP were selected for PLSR modeling. Whole Raman spectra regions, the combined regions of 3150–2460 and 1800–769 cm^−1^, and regions of 1800–769 cm^−1^ provide comparable results during the calibration modeling in terms of R^2^ values, RMSECV, and number of PLS factors. This result confirmed that the combination of Raman spectra and PLSR could be used for the quantitative analysis of ω-3 FAs with acceptable accuracy and precision performances [80]. Raman spectra combined with chemometrics of PLSR and PCA could be used for quantitative analysis of EPA, DHA, and total ω-3 FAs and classification of fish oils and ω-3 PUFA concentrates in intact soft gel (gelatin) capsules [81]. The handled Raman spectroscopy combined with multivariate calibrations of PLSR using the algorithm of NIPALS has been employed for analysis of EPA, DHA, and EPA + DHA in the commercial supplement samples of encapsulated omega-3 oils. The absorbance values of Raman spectra at regions of 1800 to 800 cm^−1^ were used for this task [82].

The nuclear magnetic resonance (NMR) spectrometry method could be successfully utilized for analysis of ω-3 FAs in fish oil by accurate quantification of EPA and DHA. Dimethyl terephthalate was used as an internal standard. The NMR peak at 2.391 ppm was selected for DHA quantification, whereas the peak at 1.697 was chosen for EPA quantification. The method was validated, demonstrating good results in terms of specificity, precision, and stability [83]. From the above results, it can be concluded that FTIR and Raman spectroscopy combined with suitable chemometrics techniques could be an alternative method for the official method based on GC-MS. Molecular spectroscopy offers a rapid and sensitive enough method with comparable results to GC-MS for determination of ω-3 FAs and ω-6 FAs in marine products with the main advantage of simplicity in instrument operation and with minimum sample preparation [84].

## 6. Materials and Methods

Before preparing the review, a number of published papers including review articles, original research articles, and reports were collected through several databases such as Scopus, Google Scholar, Web of Science, and Pubmed. During the literature searching, the keywords used consisted of “liquid chromatography”, “gas chromatography”, “liquid chromatography-mass spectrometry”, “gas chromatography-mass spectrometry”, “gas chromatography-flame ionization detector”, “vibrational spectroscopy”, “FTIR spectroscopy”, “NIR spectroscopy”, “Raman spectroscopy”, “omega-3 and omega-6 in marine natural products”, “analysis of omega-3 from marine natural products”, “analysis of omega-6 from marine natural products”, “partial least square regression”, “principal component regression”, “multivariate calibration”, and “chemometrics”. The searching was conducted either using separate keywords or a combination of two or more keywords. The inclusion criteria for selected papers were (1) studies regarding analysis of omega-3 and omega-6 using spectroscopic methods conducted in 2000–2023, (2) studies of omega-3 and omega-6 analysis using liquid chromatography-based methods between 1996 and 2023, (3) studies on the analysis of omega-3 and omega-6 analysis using gas chromatography-based method conducted between 2000 and 2023, (4) studies on the use of chemometrics for analysis of omega-3 and omega-6, and (5) all papers written in English. The MarvinSketch 16.2.29.0 program was used for drawing the chemical structures of molecules in this review.

## 7. Conclusions

Marine products such as fish oils are rich in ω-3 FAs and ω-6 FAs, which are believed to have beneficial roles in human health. Food supplements and pharmaceutical products contain both ω-3 FAs and ω-6 FAs in their formulations with certain levels, so the development and employment of accurate and valid analytical methods for routine quality control are very urgent. Gas chromatography coupled with flame ionization detection (GC-FID) and GC-mass spectrometry (GC-MS) are the methods of choice (official methods) for analysis of ω-3 FAs and ω-6 FAs in marine-based products, especially fish oils. In addition, molecular spectroscopy such as Infrared and Raman in combination with chemometrics has been successfully applied for quantitative analysis of individual and total ω-3 FAs and ω-6 FAs and is considered as an alternative technique to GC-FID and GC-MS. Gas chromatography and liquid chromatography-based methods provide advantages in terms of sensitivity for analysis of ω-3 FAs and ω-6 FAs in marine natural products. However, a simplification of sample preparation could be acquired using molecular spectroscopy techniques. Therefore, in the future, molecular spectroscopic techniques should be subjected to collaborative study among competent laboratories to prove that molecular spectroscopy is one of the standard methods for analysis of ω-3 FAs and ω-6 FAs in marine natural products as well as in food and pharmaceutical products.

## Figures and Tables

**Table 1:** The application of GC-FID and GC-MS for analysis of ω-3 and ω-6 FAs in fish oils contained in numerous products.

ω-3 and ω-6 FAs (Samples)	Sample Preparation	GC Condition	Results	Ref.
ALA and DHA in mackerel fish oil	The fish was subjected to boiling, and the separated oils were extracted using ethanol-chloroform. Fish oils were subjected to derivatization to provide FAMEs with BF_3._	Capillary column with DB-5 stationary phase (30 m × 0.32 mm, 0.25 μm film thickness), carrier gas: helium; splitness; oven was programmed at temperature 160–200 °C with flow rate 1.0, detector FID and the injector temperatures were set at 250 °C.	APA and DHA were well separated with good efficiency as indicated with low high equivalent theoretical plate. The recovery obtained was in the range of 100.36 ± 0.31 (APA) to 100.58 ± 0.32. The levels of APA and DHA found were 0.181–0.214% (APA) and 0.010–0.321%.	[51]
EPA and DHA in fish oil capsules	The fish oil was taken from fish oil capsules then used for derivatization of the polyunsaturated fatty acids into FAMEs.	Capillary column of nitroterephthalic acid-modified polyethylene glycol, PEG bonded (30 m × 0.32 mm ID × 0.25 µm), carrier gas: helium, oven temperature was programmed at temperature of 100–240 °C with total running time of 38 min using flow rate of 0.8 mL/min; injector temperature was 240 °C in split mode (20:1); the detector temperature (ion trap mass spectrometer) was 240 °C.	The combined concentrations of EPA and DHA ranged from 160.6–360.4 mg/g, with an average result of 197.3 ± 50.7 mg/g. The limit of detection and limit of quantification were 0.16–0.18 mg/g and 0.46–0.63 mg/g, respectively, with recoveries above 76%.	[52]
EPA and DHA in fish oil capsules	The fish oil from fish oil capsules was pipetted, weighed, and subjected to derivatization to form FAMEs using BF_3._	Capillary column of RTX-5SM (60 m × 0.25 mm, layer thickness 0.25 μm), carrier gas: helium with flow rate of 0.73 mL/min, oven temperature was programmed from 80–280 °C, injector temperature was 250 °C with split ratio 1:200, the temperature of the electron impact source was set at 200 °C	The EPA and DHA in fish oil capsules were determined in relative areas. All tested products had relative content of EPA and DHA in accordance with the respective labels.	[53]
EPA and DHA in fish oil nutritional capsules	The fish oil nutritional samples were subjected to derivatization to obtain FAMEs using BF_3._	A DB5-MS capillary column (30 m × 0.32 mm ID × 0.25 µm) was used, carrier gas: helium (0.8 mL/min), oven temperature was programmed at 80–280 °C, injector temperature was 250 °C with a split ratio of 10:1, ion source temperature of EI was 200 °C.	The GC-MS method had good sensitivity, accuracy, and precision. The LoDs for EPA and DHA were 0.08 ng and 0.21 ng, respectively. The content of EPA was 39.52 to 509.16 mg/g, and the content of DHA was 35.14 to 645.70 mg/g. The obtained recovery was 100.50% and 103.83% for EPA and DHA, respectively, with RSD less than 1.05% for both EPA and DHA.	[49]
EPA and DHA in fish	Fish samples were divided into three groups: raw, baking, and steaming. The fat content was extracted using the Bligh and Dyer method. The obtained fats were then subjected to derivatization to provide FAMEs using BF_3._	A capillary column with high polarity of GC HP-88 (60 m length, 0.25 mm ID, 0.2 µm DF) was used, carrier gas: helium with flow rate of 1 mL/min, the oven temperature was programmed from 40 °C to 230 °C, the temperature of the injector was 250 °C with a split ratio of 20:1, the FID temperature was 250 °C.	The method could separate EPA and DHA with good resolution. The method had a high accuracy shown by the recovery (>95%) and good precision (RSD ≤ 2%).	[48]
EPA and DHA in commercial omega-3 dietary supplements	Supplements of cod liver oils, algal oils, krill oils, and fish oils were subjected to derivatization to obtain FAMEs using BF_3._	A capillary column of wax CP-52CB (CP 8843, 30 m × 0.32 mm I.D., DF-25 coating thickness 0.25 μm) was used. The oven temperature was programmed from 170–240 °C. Carrier gas was helium at a flow rate of 2.5 mL/min. The temperature of the injector was set at 250 °C, whereas FID was operated at 300 °C.	The contents of EPA and DHA for fish and krill-based supplements were 81.8–456.4 mg/g oil and 51.6–220.4 mg/g oil, respectively. For algal oil, the content of EPA was 7.7–151.1 mg/g and the content of DHA was 237.8–423.5 mg/g oil.	[54]
EPA and DHA in South Pacific fish and shellfish species	Samples were subjected to total lipids extraction applying the Folch method then used for the derivatization of the fatty acids to obtain FAMEs using BF_3_.	Analysis was performed using GC-FID using programmed temperature with helium as the carrier gas.	Red cusk eel contained EPA and DHA levels of 40.8 and 74.4 mg/100 g, respectively. Mackerel contained 414.7 and 956.0 mg/100 g of EPA and DHA, respectively. Sea squirt (shellfish species) contained EPA and DHA levels of 375.0 and 165.7 mg/100 g, respectively. In addition, EPA + DHA content in Chilean abalone was 63.6 mg/100 g.	[55]
EPA and DHA in Irish microalgal isolates (three marine strains: diatom *cf*. *Stauroneis* sp. LACW24, chrophyte *cf*. *Phaeothamnion* sp. LACW34, and haptophyte *Diacronema* sp. GMC30)	The total lipids of microalgal isolates were extracted using the Bligh and Dyer method. The lipids were further processed and used in derivatization to obtain FAMEs using BF_3._	A capillary column of BPX70 120 m length and 0.22 mm internal diameter was used. The oven temperature was programmed from 50–240 °C. The carrier gas was helium operated at 2 mL/min. The sample was injected at a split ratio of 100:1 at an injector temperature of 250 °C. The detector temperature of the MS source was set at 230 °C, and the MS Quad was at 150 °C.	The average yields of EPA were 3.9, 11.9, and 1.3 mg EPA/g DW for GMC30, LACW24, and LACW34, respectively. The average yields of DHA were 3.0 and 2.0 mg DHA/g DW for GMC30 and LACW34, respectively.	[56]
EPA and DHA in marine fish species in Turkish waters	The total lipids from twelve marine fish species in Turkey were extracted, and the fatty acids content was further derivatized to provide FAMEs.	A fused silica capillary column (25 m × 0.2 mm ID) was used, and the oven temperature was set from 170–300 °C. The carrier gas was hydrogen. The injector temperature was 250 °C, whereas the detector temperature (FID) was 300 °C.	DHA ranged from 43.7 to 75.2%. The n-3/n-6 FA ratio ranged from 2.67 to 12.61.	[57]
EPA and DHA in sardine oil	The lipids of sardine oil were subjected to derivatization to obtain FAMEs.	Analysis was performed using a capillary column (EC-wax, 30 m × 0.25 mm i.d.) at oven temperature programmed from 50 °C to 220 °C. The split ratio of sample injection was 100:1 with an injector temperature of 250 °C. The detector (FID) temperature was set at 270 °C.	The unhydrolyzed oil contained EPA and DHA levels of 26.86% and 13.62%, respectively. The hydrolyzed oil contained 33.74% EPA and 29.94% DHA.	[58]

**Table 2:** The application of HPLC and LC-MS for analysis of ω-3 and ω-6 FAs in marine products.

ω-3 and ω-6 FAs (Samples)	Sample Preparation	HPLC/LC-MS or LC-MS/MS Condition	Results	Ref.
EPA and DHA from fish oils (rainbow trout oil)	The fatty acids were extracted, then the EPA and DHA were further purified using HPLC.	HPLC method using mobile phase of ethanol HPLC grade and ultra-pure water. Elution of 5 µL injected sample was performed for 20 min and detected using UV detector at 254 nm.	The fractions of EPA and DHA could be separated from other fatty acids after purification and could be detected using the HPLC-UV system. The retention time was between 3.5 and 4.7 min.	[63]
DHA from marine microalgae (*Schizochytrium* sp. SH103)	The lipids were extracted from dried cells using chloroform: methanol (2:1 *v*/*v*) for 20 min. The chloroform layer was taken and concentrated using a rotary evaporator. After that, the extracted lipids were converted into fatty acids ethyl esters (FAEEs) using acid-catalyzed transesterification.	Semi-preparative HPLC with UV detector. Reverse-phase system (C18 column 250 mm, 4.6 mm, 2.0 µm). Two small columns were connected using a connector with dead volume of 20 µL in order to optimize the semi-preparative condition. Methanol at various concentrations was used as the mobile phase.	The optimum separation of DHA was obtained using the isocratic condition employing the mobile phase of methanol/water (96:4 *v*/*v*) with velocity of 0.5 mL/min. The purity of DHA was 98.5%.	[64]
EPA and DHA from tuna fish oils	The heads, skins, fishbones, and gullets of tuna fish were used for fish oil extraction. The homogenate was added with distilled water at a ratio of 1:1 then heated for 60 min at 50 °C. The oil phase was separated from the water layer and used for analysis.	LC-APCI/MS was used, equipped with a Hypersil column (50 mm, 2.1 mm, 1.8 µm), and the temperature was maintained at 25 °C. Analysis was performed in positive ionization with APCI parameters of corona probe current: 4 µA, corona voltage: 3.6 kV, and probe temperature: 450.0 °C.	EPA and DHA could be detected using HPLC/HRMS along with other TAG compounds.	[65]
EPA and DHA in several aquatic products	The lipids were extracted from each aquatic product using solvent extraction techniques.	HILIC-MS/MS method using specific precursor ion scanning.	The total identified PL_EPA/DHA_ molecules were 80. The best resource for PL_EPA/DHA_ was Antarctic krill (2574.69 µg/g), followed by mackerel (2330.11 µg/g), salmon (2109.91 µg/g), and Farrer’s scallop (1883.59 µg/g). Sea cucumber contained the highest contents of EPA-structured phospholipids, whereas sea bass contained the highest DHA-structured phospholipids.	[66]
EPA from marine microalgae of *Phaeodactylum tricornutum*	Three step preparation process: extraction of fatty acids (direct saponification of biomass), PUFA concentration, and EPA isolation.	A semi-preparative HPLC with reverse-phase system using a C18 column 25 cm × 10 mm i.d.. The mobile phase was methanol:water 80:20 *v*/*v* containing 1% acetic acid eluted in isocratic mode.	An amount of 65.7% EPA present in biomass was recovered in highly pure form. In addition, without the PUFA concentration step, 93.6% EPA could be isolated from pure fatty acid extracts.	[67]
EPA and DHA in fish oils	The fish oil was extracted using n-hexane and used for esterification in ethanol to obtain fatty acid ethyl esters. Subsequently, the purification was conducted to obtain concentrated EPA ethyl ester (EPA-EE) and DHA ethyl ester (DHA-EE).	An HPLC binary pump connected to APCI-MS was used. Analysis was performed using an AgTCM (150 mm × 3.0 mm i.d., 3 μm) column set at 25 °C. For APCI-MS parameters: vaporization temperature was 400 °C; nebulization pressure was 60 psig; drying gas flow and temperature were 5.0 L/min and 300 °C; corona current of 3.5 μA with capillary voltage of 3500 V.	The EPA-EE and DHA-EE could be separated from other fatty acid peaks, and the purification process could yield EA-EE and DHA-EE with purity above 95%.	[68]
EPA and DHA in capelin (*Mallotus villosus*) fish	Crude fat was extracted according to the AOAC method. The lipids were extracted using chloroform:methanol (2:1, −20 °C).	LC-HRMS Orbitrap using a C18 column (100 mm × 2.1 mm × 1.7 µm) set at a temperature of 55 °C. The mobile phase used was acetonitrile:water (60:40) (A) and isopropanol:acetonitrile (90:10) (B) both of them containing 10 mM ammonium formate. Elution was performed in gradient mode for 18 min.	The content of EPA was 7.79–16.91%, whereas the content of DHA was 7.65–19.83%.	[69]
ω-3 FAs in fish oils	Fish oils were subjected to oxidation then used for solid phase extraction (SPE) for both oxidized and non-oxidized forms.	LC-MS/MS using a reverse-phase system (C18 column, 100 mm, 5 mm, 1.8 µm) with water and acetonitrile containing 0.1% formic acid as mobile phases. Analytes were eluted using gradient mode with running time 35 min. The MS detector was a triple quadrupole MS operated at negative ionization mode. The temperature for the ion transfer tube was 325 °C with vaporizer temperature of 150 °C.	The LC-MS/MS method could distinguish carbonyl species from omega-3 and omega-6 fatty acids. The validated method could be applied to monitor the formation of carbonyl species in different fish oils caused by lipid peroxidation.	[70]
ω-3 FAs and ω-6 FAs in golden threadfin bream (*Nemipterus virgatus*) fish	Lipids were extracted using the Folch method and redissolved in methanol:isopropanol (1:1 *v*/*v*) then added with internal standard of phosphatidylethanolamine (15:0–18:1-d7-PE).	LC-Orbitrap HRMS using a reverse-phase system (C18 column 100 mm, 2.1 mm, 1.7 µm). The mobile phase was acetonitrile:water (60:40) and acetonitrile:propanol (10:90) both containing 10 mM ammonium formate. The MS detector was operated in both (+) and (−) ionization modes with capillary temperature 320 °C in ESI+ and 300 °C in ESI−. The resolution was 70,000 (full MS) and 17,500 (MS/MS).	High contents of phospholipids and saccharolipids were observed. The EPA, DHA, and ARA (arachidonic acid) PUFA were found to be dominant. The EPA content was 3.89–5.29%, the DHA content was 11.07–21.54%, and the ARA content was 2.36–3.64%.	[71]
EPA and DHA from *Decapterus maruadsi* fish	Fish samples were freeze dried, then the lipids were extracted using a modified Folch method. Isopropanol:methanol 1:1 *v*/*v* was used to resuspend the lipids.	The reverse-phase LC system using a C18 column (BEH 100 mm, 2.1 mm, 1.7 µm) was used. The mobile phase was acetonitrile:water (60:40) and acetonitrile:propanol (10:90) both containing ammonium formate (10 mM) and acetic acid (0.1%). The detection was performed using Orbitrap HRMS operated at positive and negative ionization modes with capillary temperature set at 320 °C and the scan range of 150–2000 *m*/*z*.	Higher proportions of EPA and DHA were found. The content of EPA was 148.84 ± 18.52 mg/100 g raw sample, whereas the content of DHA was 384.30 ± 17.67 mg/100 g raw sample.	[72]

## Data Availability

Data is contained within the article.

## Abbreviations

APCI-MS	Atmospheric pressure chemical ionization-mass spectrometry
ATR	Attenuated total reflectance
^13^C-NMR	Proton nuclear magnetic resonance
CVD	Cardiovascular diseases
DHA	Docosahexaenoic acid
ECD	Electrochemical detector
EFSA	European Food Safety Authority
EPA	Eicosapentaenoic acid
FAO	Food and Agriculture Organization
FAs	Fatty acids
FAMEs	Fatty acids methyl
FTIR	Fourier Transform Infrared
GC-FID	Gas chromatography-flame ionization detector
GC-MS	Gas chromatography-mass spectrometry
^1^H-NMR	Proton nuclear magnetic resonance
HP-88	High polarity-88
ICH	International Council for Harmonization
HPLC	High-performance liquid chromatography
LC–MS/MS	Liquid chromatography tandem mass spectrometry
LDA	Linear discriminant analysis
MHz	Megahertz
PCA	Principal component analysis
PCR	Principal component regression
PE-FFAP	Nitroterephthalic acid-modified polyethylene glycol, PEG bonded
PEG	Polyethylene glycol
PLSR	Partial least square regression
PUFA	Polyunsaturated fatty acid
R^2^	Coefficient of determination
RMSEC	Root mean square error of calibration
RMSEP	Root mean square error of prediction
RRt	Relative retention time
Rt	Retention time
SIMCA	Soft independent modeling class analogy
SST	Standard solution test
TAGs	Triacylglycerols
TGs	Triglycerides
UPLC	Ultra-performance liquid chromatography
UV	Ultraviolet
WHO	World Health Organization
ω-3	Omega-3
ω-6	Omega-6

## References

[1] Simopoulos A.P. (2010). The omega-6/omega-3 fatty acid ratio: Health implications. OCL Ol. Corps Gras Lipides.

[2] Maruba P., Jamaran K., Basuki W., Jansen S. (2018). Determination and identification of omega 3 and 6 fatty acids position in nile tilapia oil. IOP Conf. Ser. Earth Environ. Sci..

[3] Brown T.J., Brainard J., Song F., Wang X., Abdelhamid A., Hooper L. (2019). Omega-3, omega-6, and total dietary polyunsaturated fat for prevention and treatment of type 2 diabetes mellitus: Systematic review and meta-analysis of randomised controlled trials. BMJ.

[4] Khalid W., Gill P., Arshad M.S., Ali A., Ranjha M.M.A.N., Mukhtar S., Afzal F., Maqbool Z. (2022). Functional behavior of DHA and EPA in the formation of babies brain at different stages of age, and protect from different brain-related diseases. Int. J. Food Prop..

[5] Balić A., Vlašić D., Žužul K., Marinović B., Mokos Z.B. (2020). Omega-3 versus Omega-6 polyunsaturated fatty acids in the prevention and treatment of inflammatory skin diseases. Int. J. Mol. Sci..

[6] Burdge G.C., Calder P.C. (2005). Conversion of α-linolenic acid to longer-chain polyunsaturated fatty acids in human adults. Reprod. Nutr. Dev..

[7] Nishiyama M.F., de Souza A.H.P., Gohara A.K., dos Santos H.M.C., de Oliveira C.A.L., Ribeiro R.P., de Souza N.E., Gomes S.T.M., Matsushita M. (2014). Chemometrics applied to the incorporation of omega-3 in tilapia fillet feed flaxseed flour. Food Sci. Technol..

[8] Lin Y.H., Hibbeln J.R., Domenichiello A.F., Ramsden C.E., Salem N.M., Chen C.T., Jin H., Courville A.B., Majchrzak-Hong S.F., Rapoport S.I. (2018). Quantitation of Human Whole-Body Synthesis-Secretion Rates of Docosahexaenoic Acid and Eicosapentaenoate Acid from Circulating Unesterified α-Linolenic Acid at Steady State. Lipids.

[9] Hadley K.B., Ryan A.S., Forsyth S., Gautier S., Salem N. (2016). The essentiality of arachidonic acid in infant developments. Nutrients.

[10] Rincón-Cervera M.Á., González-Barriga V., Romero J., Rojas R., López-Arana S. (2020). Quantification and distribution of omega-3 fatty acids in South Pacific fish and shellfish species. Foods.

[11] Djuricic I., Calder P.C. (2021). Beneficial outcomes of omega-6 and omega-3 polyunsaturated fatty acids on human health: An update for 2021. Nutrients.

[12] Brereton R.G. (2015). Pattern recognition in chemometrics. Chemom. Intell. Lab. Syst..

[13] Brereton R.G., Jansen J., Lopes J., Marini F., Pomerantsev A., Rodionova O., Roger J.M., Walczak B., Tauler R. (2018). Chemometrics in analytical chemistry—Part II: Modeling, validation, and applications. Anal. Bioanal. Chem..

[14] Riswanto F.D.O., Windarsih A., Lukitaningsih E., Rafi M., Fadzilah N.A., Rohman A. (2022). Metabolite Fingerprinting Based on ^1^H-NMR Spectroscopy and Liquid Chromatography for the Authentication of Herbal Products. Molecules.

[15] Sridhar K., Charles A.L. (2018). Application of multivariate statistical techniques to assess the phenolic compounds and the in vitro antioxidant activity of commercial grape cultivars. J. Chemom..

[16] Irnawati I., Riswanto F.D.O., Riyanto S., Martono S., Rohman A. (2021). The use of software packages of R factoextra and FactoMineR and their application in principal component analysis for authentication of oils. Indones. J. Chemom. Pharm. Anal..

[17] Zhang X.Y., Hu W., Teng J., Peng H.H., Gan J.H., Wang X.C., Sun S.Q., Xu C.H., Liu Y. (2017). Rapid recognition of marine fish surimi by one-step discriminant analysis based on near-infrared diffuse reflectance spectroscopy. Int. J. Food Prop..

[18] Alamprese C., Casiraghi E. (2015). Application of FT-NIR and FT-IR spectroscopy to fish fillet authentication. LWT.

[19] Nieto-Ortega S., Olabarrieta I., Saitua E., Arana G., Foti G., Melado-Herreros Á. (2022). Improvement of Oil Valorization Extracted from Fish By-Products Using a Handheld near Infrared Spectrometer Coupled with Chemometrics. Foods.

[20] Jahani R., Yazdanpanah H., van Ruth S.M., Kobarfard F., Alewijn M., Mahboubi A., Faizi M., Aliabadi M.H.S., Salamzadeh J. (2020). Novel application of near-infrared spectroscopy and chemometrics approach for detection of lime juice adulteration. Iran. J. Pharm. Res..

[21] Devos O., Downey G., Duponchel L. (2014). Simultaneous data pre-processing and SVM classification model selection based on a parallel genetic algorithm applied to spectroscopic data of olive oils. Food Chem..

[22] Biancolillo A., Marini F., Ruckebusch C., Vitale R. (2020). Chemometric Strategies for Spectroscopy-Based Food Authentication. Appl. Sci..

[23] Karunathilaka S.R., Choi S.H., Mossoba M.M., Yakes B.J., Brückner L., Ellsworth Z., Srigley C.T. (2019). Rapid classification and quantification of marine oil omega-3 supplements using ATR-FTIR, FT-NIR and chemometrics. J. Food Compos. Anal..

[24] *USP 46—NF 41*; Monograph, Dietary Supplement: Omega-3 Free Fatty Acids.

[25] *USP 46—NF 41*; Monograph, Dietary Supplement: Omega-3 Acid Triglycerides.

[26] *USP 46—NF 41*; Monograph, Dietary Supplement: Omega-3-Acid Ethyl Esters.

[27] Alaerts G., Van Erps J., Pieters S., Dumarey M., van Nederkassel A.M., Goodarzi M., Smeyers-Verbeke J., Vander Heyden Y. (2012). Similarity analyses of chromatographic fingerprints as tools for identification and quality control of green tea. J. Chromatogr. B.

[28] *USP 46—NF 41*; General chapter <401> Fats and Fixed Oils.

[29] *USP 46—NF 41*; Monograph, Dietary Supplement: Fish Oil Containing Omega-3 Acids.

[30] *USP-NF, USP44-NF39*; <1226> Verification of Compendial Procedures. https://online.uspnf.com/uspnf/document/1_GUID-18A6E56B-8EA7-4B37-AB7D-82352B73A309_3_en-US.

[31] *USP-NF, USP44-NF39*; <1225> Validation of Compendial Procedures. https://online.uspnf.com/uspnf/document/1_GUID-E2C6F9E8-EA71-4B72-A7BA-76ABD5E72964_4_en-US.

[32] Viswanathan S., Verma P.R.P., Ganesan M., Manivannan J. (2017). A novel liquid chromatography/tandem mass spectrometry (LC–MS/MS) based bioanalytical method for quantification of ethyl esters of Eicosapentaenoic acid (EPA) and Docosahexaenoic acid (DHA) and its application in pharmacokinetic study. J. Pharm. Biomed. Anal..

[33] Zeng Y.X., Araujo P., Du Z.Y., Nguyen T.T., Frøyland L., Grung B. (2010). Elucidation of triacylglycerols in cod liver oil by liquid chromatography electrospray tandem ion-trap mass spectrometry. Talanta.

[34] European Pharmacopoeia 11.1. 2.2.28. Gas Chromatography. https://www.google.com.ua/url?sa=t&rct=j&q=&esrc=s&source=web&cd=&ved=2ahUKEwiXxar5touAAxVNwjgGHY4gD8QQFnoECAsQAQ&url=https%3A%2F%2Ffile.wuxuwang.com%2Fyaopinbz%2FEP9%2FEP9.0_01__37.pdf&usg=AOvVaw0CKj2kZCcgPpt6R9bBYbQG&opi=89978449.

[35] *USP 46—NF 41*; General Chapter <621> Chromatography. https://www.usp.org/sites/default/files/usp/document/harmonization/gen-chapter/harmonization-november-2021-m99380.pdf.

[36] Yuwono M., Indrayanto G., Cazes J. (2004). GC system instrumentation. Dekker Encyclopedia of Chromatography.

[37] Scott R.P.W. (2022). Gas Chromatography–Mass Spectrometry Systems. Encyclopedia of Chromatography.

[38] Harvey D.J. (2021). Mass spectrometric detectors for gas chromatography. Gas Chromatogr..

[39] Yuwono M., Indrayanto G., Cazes J. (2004). On-Column Injection for GC. Encyclopedia of Chromatography, Update Supplement.

[40] Swinley J., Coning P. (2019). de A Practical Guide to Gas Analysis by Gas Chromatography.

[41] Yuwono M., Indrayanto G., Cazes J. (2005). Gas Chromatography. Ewing’s Analytical Instrumentation Handbook.

[42] Noviana E., Indrayanto G., Rohman A. (2022). Advances in Fingerprint Analysis for Standardization and Quality Control of Herbal Medicines. Front. Pharmacol..

[43] Indrayanto G. (2022). The importance of method validation in herbal drug research. J. Pharm. Biomed. Anal..

[44] Indrayanto G. (2018). Validation of Chromatographic Methods of Analysis: Application for Drugs That Derived From Herbs. Profiles of Drug Substances, Excipients and Related Methodology.

[45] Tang B., Row K.H. (2013). Development of gas chromatography analysis of fatty acids in marine organisms. J. Chromatogr. Sci..

[46] Petrović M., Kezić N., Bolanča V. (2010). Optimization of the GC method for routine analysis of the fatty acid profile in several food samples. Food Chem..

[47] Juárez M., Juárez A., Aldai N., Avilés C., Polvillo O. (2010). Validation of a gas-liquid chromatographic method for analysing samples rich in long chain n-3 polyunsaturated fatty acids: Application to seafood. J. Food Compos. Anal..

[48] Alinafiah S.M., Azlan A., Ismail A., Rashid N.K.M.A. (2021). Method development and validation for omega-3 fatty acids (Dha and epa) in fish using gas chromatography with flame ionization detection (gc-fid). Molecules.

[49] Yi T., Li S.M., Fan J.Y., Fan L.L., Zhang Z.F., Luo P., Zhang X.J., Wang J.G., Zhu L., Zhao Z.Z. (2014). Comparative analysis of EPA and DHA in fish oil nutritional capsules by GC-MS. Lipids Health Dis..

[50] Wang P., Sun M., Ren J., Djuric Z., Fisher G.J., Wang X., Li Y. (2017). Gas chromatography-mass spectrometry analysis of effects of dietary fish oil on total fatty acid composition in mouse skin. Sci. Rep..

[51] Arfan T., Harmita, Maggadani B.P. (2018). Analysis of alpha-linolenic acid and docosahexaenoic acid in mackerel fish oil (Rastrelliger Kanagurta) using gas chromatography. Int. J. Appl. Pharm..

[52] Brotas M.S.C., Carvalho G.A., Pereira P.A.P. (2020). Determination, through Derivatization and GC-MS Analysis, of Omega-3 and Omega-6 Fatty Acids in Fish Oil Capsules Sold in Salvador, Bahia. J. Braz. Chem. Soc..

[53] Lorensia A., Budiono R., Suryadinata R.V., Tiarasari N. (2021). Quantitative determination of EPA and DHA in fish oil capsules for cardiovascular disease therapy in Indonesia by GC-MS. J. Public health Res..

[54] Kleiner A.C., Cladis D.P., Santerre C.R. (2015). A comparison of actual versus stated label amounts of EPA and DHA in commercial omega-3 dietary supplements in the United States. J. Sci. Food Agric..

[55] Rincón-Cervera M.Á., Villarreal-Rubio M.B., Valenzuela R., Valenzuela A. (2017). Comparison of fatty acid profiles of dried and raw by-products from cultured and wild fishes. Eur. J. Lipid Sci. Technol..

[56] Archer L., Mc Gee D., Paskuliakova A., McCoy G.R., Smyth T., Gillespie E., Touzet N. (2019). Fatty acid profiling of new Irish microalgal isolates producing the high-value metabolites EPA and DHA. Algal. Res..

[57] Bayir A., Haliloǧlu H.I., Sirkecioǧlu A.N., Aras N.M. (2006). Fatty acid composition in some selected marine fish species living in Turkish waters. J. Sci. Food Agric..

[58] Okada T., Morrissey M.T. (2007). Production of n − 3 polyunsaturated fatty acid concentrate from sardine oil by lipase-catalyzed hydrolysis. Food Chem..

[59] European Pharmacopoeia 11.1, 2.2.29. Liquid Chromatography. https://www.google.com.ua/url?sa=t&rct=j&q=&esrc=s&source=web&cd=&ved=2ahUKEwi3m8S-touAAxWw-jgGHdZKAr8QFnoECBQQAQ&url=https%3A%2F%2Ffile.wuxuwang.com%2Fyaopinbz%2FEP9%2FEP9.0_01__38.pdf&usg=AOvVaw0swpPGkWRzIbNCaU6wOL_q&opi=89978449.

[60] (2021). Merck KGaA. A Practical Guide to High Performance Liquid Chromatography. https://app.go.sigmaaldrich.com/e/er?utm_campaign=AP_AA_MRK_2023028066_48188_Pharma%20Chem_MAY23_Em%202&utm_medium=email&utm_source=Eloqua&s=832461399&lid=32798&elqTrackId=b0b25b4bdea04c1fbc49b43ac2cd2be7&elq=f5d245faaca04384bb2503404f50d98b&elqaid=44691&elqat=1.

[61] The Ministry of Health, Labour, and Welfare (2021). The Japanese Pharmacopoeia.

[62] Steiner D., Krska R., Malachová A., Taschl I., Sulyok M. (2020). Evaluation of Matrix Effects and Extraction Efficiencies of LC-MS/MS Methods as the Essential Part for Proper Validation of Multiclass Contaminants in Complex Feed. J. Agric. Food Chem..

[63] Ucar Y., Ozogul F., Durmus M., Ozogul Y., Kosker A.R., Boga E.K., Ayas D. (2019). Purification of eicosapentaenoic acid (EPA) and docosahexaenoic acid (DHA) from fish oil using HPLC method and investigation of their antibacterial effects on some pathogenic bacteria. Turkish J. Marit. Mar. Sci..

[64] Oh C.E., Kim G.J., Park S.J., Choi S., Park M.J., Lee O.M., Seo J.W., Son H.J. (2020). Purification of high purity docosahexaenoic acid from *Schizochytrium* sp. SH103 using preparative-scale HPLC. Appl. Biol. Chem..

[65] Indelicato S., Di Stefano V., Avellone G., Piazzese D., Vazzana M., Mauro M., Arizza V., Bongiorno D. (2023). HPLC/HRMS and GC/MS for Triacylglycerols Characterization of Tuna Fish Oils Obtained from Green Extraction. Foods.

[66] Xue J., Ge L., Wang H., Liang J., Wang Q., Lu W., Cui Y., Xie H., Jian S., Jin D. (2023). Comprehensive Screening for EPA/DHA-Structured Phospholipids in Aquatic Products by a Specific Precursor Ion Scanning-Based HILIC-MS/MS Method. J. Agric. Food Chem..

[67] Cartens M., Molina Grima E., Robles Medina A., Giménez Giménez A., Ibánez Gonzalez J. (1996). Eicosapentaenoic acid (20:5n-3) from the Marine Microalga *Phaeodactylum tricornutum*. J. Am. Oil Chem. Soc..

[68] Dillon J.T., Aponte J.C., Tarozo R., Huang Y. (2013). Purification of omega-3 polyunsaturated fatty acids from fish oil using silver-thiolate chromatographic material and high performance liquid chromatography. J. Chromatogr. A.

[69] Yin M., Chen M., Matsuoka R., Song X., Xi Y., Zhang L., Wang X. (2023). UHPLC-Q-Exactive Orbitrap MS/MS based untargeted lipidomics reveals fatty acids and lipids profiles in different parts of capelin (*Mallotus villosus*). J. Food Compos. Anal..

[70] Suh J.H., Niu Y.S., Hung W.L., Ho C.T., Wang Y. (2017). Lipidomic analysis for carbonyl species derived from fish oil using liquid chromatography–tandem mass spectrometry. Talanta.

[71] Cui J., Cao J., Zeng S., Ge J., Li P., Li C. (2022). Comprehensive evaluation of lipidomics profiles in golden threadfin bream (*Nemipterus virgatus*) and its by-products using UHPLC-Q-exactive Orbitrap-MS. LWT.

[72] He C., Sun Z., Qu X., Cao J., Shen X., Li C. (2020). A comprehensive study of lipid profiles of round scad (*Decapterus maruadsi*) based on lipidomic with UPLC-Q-Exactive Orbitrap-MS. Food Res. Int..

[73] Acworth I., Plante M., Crafts C., Bailey B. (2011). Quantitation of Underivatized Omega-3 and Omega-6 Fatty Acids in Foods by HPLC and Charged Aerosol Detection. Planta Med..

[74] Sprynskyy M., Monedeiro F., Monedeiro-Milanowski M., Nowak Z., Krakowska-Sieprawska A., Pomastowski P., Gadzała-Kopciuch R., Buszewski B. (2022). Isolation of omega-3 polyunsaturated fatty acids (eicosapentaenoic acid—EPA and docosahexaenoic acid—DHA) from diatom biomass using different extraction methods. Algal Res..

[75] Serafim V., Tiugan D.A., Andreescu N., Mihailescu A., Paul C., Velea I., Puiu M., Niculescu M.D. (2019). Development and validation of a LC–MS/MS-based assay for quantification of free and total omega 3 and 6 fatty acids from human plasma. Molecules.

[76] Rohman A., Windarsih A. (2020). The application of molecular spectroscopy in combination with chemometrics for halal authentication analysis: A review. Int. J. Mol. Sci..

[77] Vongsvivut J., Heraud P., Zhang W., Kralovec J.A., McNaughton D., Barrow C.J. (2012). Quantitative determination of fatty acid compositions in micro-encapsulated fish-oil supplements using Fourier transform infrared (FTIR) spectroscopy. Food Chem..

[78] Amorim T.L., de la Fuente M.A., de Oliveira M.A.L., Gómez-Cortés P. (2021). ATR-FTIR and Raman Spectroscopies Associated with Chemometrics for Lipid Form Evaluation of Fish Oil Supplements: A Comparative Study. ACS Food Sci. Technol..

[79] Prado E., Eklouh-Molinier C., Enez F., Causeur D., Blay C., Dupont-Nivet M., Labbé L., Petit V., Moreac A., Taupier G. (2022). Prediction of fatty acids composition in the rainbow trout *Oncorhynchus mykiss* by using Raman micro-spectroscopy. Anal. Chim. Acta.

[80] Bekhit M.Y., Grung B., Mjøs S.A. (2014). Determination of omega-3 fatty acids in fish oil supplements using vibrational spectroscopy and chemometric methods. Appl. Spectrosc..

[81] Killeen D.P., Marshall S.N., Burgess E.J., Gordon K.C., Perry N.B. (2017). Raman Spectroscopy of Fish Oil Capsules: Polyunsaturated Fatty Acid Quantitation Plus Detection of Ethyl Esters and Oxidation. J. Agric. Food Chem..

[82] Killeen D.P., Card A., Gordon K.C., Perry N.B. (2020). First Use of Handheld Raman Spectroscopy to Analyze Omega-3 Fatty Acids in Intact Fish Oil Capsules. Appl. Spectrosc..

[83] Lv J., Wang C., Zhang X., Zhihua L., Yu M. (2020). ^1^H-NMR quantification of DHA and EPA in fish oil. J. Ocean Univ. China..

[84] Moros J., Garrigues S., Guardia M. (2010). de la Vibrational spectroscopy provides a green tool for multi-component analysis. TrAC—Trends Anal. Chem..

